# Does fragmented cancer care affect survival? Analysis of gastric cancer patients using national insurance claim data

**DOI:** 10.1186/s12913-022-08988-y

**Published:** 2022-12-21

**Authors:** Dong-Woo Choi, Sun Jung Kim, Dong Jun Kim, Yoon-Jung Chang, Dong Wook Kim, Kyu-Tae Han

**Affiliations:** 1 Cancer Big Data Center, National Cancer Control Institute, National Cancer Center, Gyeonggi-do, Goyang-Si, Republic of Korea; 2 Department of Health Administration and Management, College of Medical Science, Soonchunhyang University, Asan-Si, Republic of Korea; 3 Graduate School of Public Health and Healthcare Management, The Catholic University of Korea, Seoul, Republic of Korea; 4 Division of Cancer Control and Policy, National Cancer Control Institute, National Cancer Center, Gyeonggi-do, Goyang-Si, Republic of Korea; 5 Department of Information and Statistics, RINS, Gyeongsang National University, 501 Jinju-daero, Jinju-si, Gyeongsangnam-do South Korea

**Keywords:** Fragmented cancer care, cancer policy, Healthcare utilization, Survival

## Abstract

**Background:**

We aimed to investigate the association between fragmented cancer care in the early phase after cancer diagnosis and patient outcomes using national insurance claim data.

**Methods:**

From a nationwide sampled cohort database, we identified National Health Insurance beneficiaries diagnosed with gastric cancer (ICD-10: C16) in South Korea during 2005–2013. We analyzed the results of a multiple logistic regression analysis using the generalized estimated equation model to investigate which patient and institution characteristics affected fragmented cancer care during the first year after diagnosis. Then, survival analysis using the Cox proportional hazard model was conducted to investigate the association between fragmented cancer care and five-year mortality.

**Results:**

Of 2879 gastric cancer patients, 11.9% received fragmented cancer care by changing their most visited medical institution during the first year after diagnosis. We found that patients with fragmented cancer care had a higher risk of five-year mortality (HR: 1.310, 95% CI: 1.023–1.677). This association was evident among patients who only received chemotherapy or radiotherapy (HR: 1.633, 95% CI: 1.005–2.654).

**Conclusions:**

Fragmented cancer care was associated with increased risk of five-year mortality. Additionally, changes in the most visited medical institution occurred more frequently in either patients with severe conditions or patients who mainly visited smaller medical institutions. Further study is warranted to confirm these findings and examine a causal relationship between fragmented cancer care and survival.

**Supplementary Information:**

The online version contains supplementary material available at 10.1186/s12913-022-08988-y.

## Background

Gastric cancer is one of the most common cancers in South Korea. According to the Cancer Registry Statistics in Korea, the crude incidence of gastric cancer was 57.4 per 100,000 in 2019, ranking third among all types of cancer, behind thyroid cancer and lung cancer; however, it ranked first from 1999 to 2018 [1]. From 2006 to 2019, the proportion of cases of gastric cancer with localized stage has increased from 81.0 to 92.0%. Moreover, in South Korea, in almost all cases, surgical treatment is performed within the first 4 months after initial diagnosis of gastric cancer [2]. Although a previous study found that 48.7% of the gastric cancer patients experienced fragmented cancer care, which is associated with inferior outcomes [3], evidence for fragmented cancer care in South Korea is lacking.

Patients with cancer commonly receive fragmented cancer care, which is defined as undergoing treatment across multiple healthcare facilities [3–5]. Previous studies have demonstrated that fragmented cancer care is associated with a reduction in overall survival, high healthcare costs, unnecessary treatments, increased time to treatment, and inferior quality of care [3, 6–8]. Patient demand is concentrated in high-volume tertiary hospitals in the capital area [9–11], where they can receive multidisciplinary therapy and centralized cancer care, which have been emphasized by the National Comprehensive Cancer Network guidelines and performed mainly at these hospitals [12, 13]. Moreover, after initial treatment, the medical staff may recommend a transfer or the treated patient may relocate to a hospital for better treatment conditions [14, 15]. Lack of coordinated cancer care between hospitals may cause delays in initiating treatment and are likely to lead to fragmented cancer care because healthcare services could not be appropriately accessed [6, 16, 17].

As discussed previously, in Korea, fragmented cancer care for patients with cancer may affect patient outcomes negatively. In terms of continuity, patient outcomes such as survival may be different. Considering the high incidence and variety of gastric cancer and the related burden on patients in Korea, we aimed to investigate the association between fragmented cancer care in the early phase after gastric cancer diagnosis and patient outcomes using national claim data.

## Methods

### Study population

**Fig. 1:**
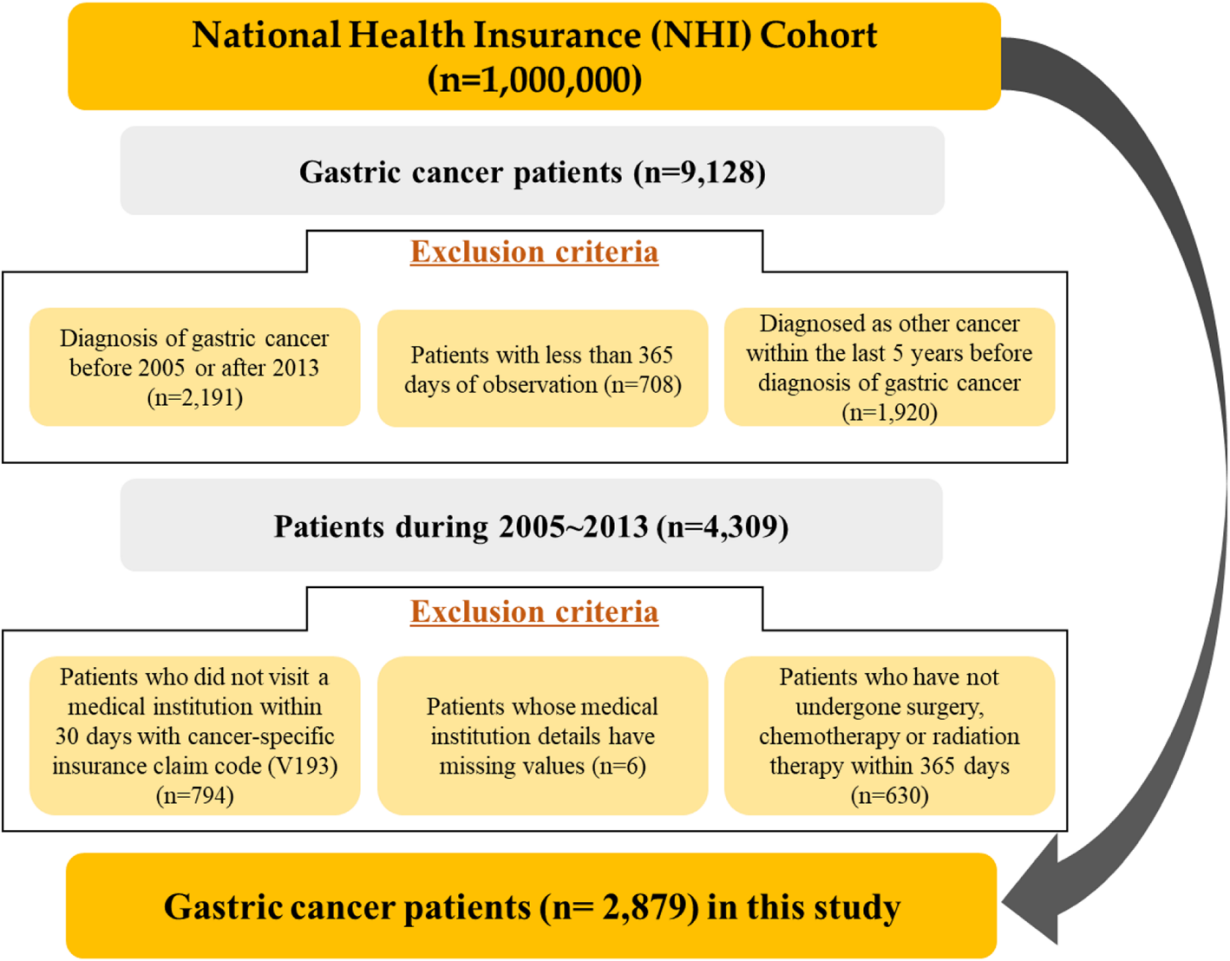
Flowchart of the study participant selection process

The data used in this study were obtained from a 2006 National Health Insurance (NHI) cohort data set comprising a sample corresponding to 2.2% (*n* = 1,000,000) of the Korean population (*N* = 48,222,537 in 2006); it was collected by stratified random sampling according to sex, age, region, types of insurance, and insurance premium. Follow-up examinations were held from 2002 to 2015 [18]. The data set included information on patient characteristics such as demographic and socioeconomic factors, healthcare utilization and treatment details, medical check-ups, and medical institution characteristics. For this study, we only included patients who had been diagnosed with gastric cancer (International Classification of Diseases [ICD]-10: C16) after 2004 or those diagnosed with other cancers in the last 5 years before gastric cancer was excluded (Fig. 1).

To reduce immortal time bias and heterogeneity among patients, only cancer patients who were diagnosed and received treatment such as surgery, chemotherapy, or radiotherapy between 2005 and 2013 were included for follow-up for at least 2 years after diagnosis, and those who died within 1 year of diagnosis were excluded. In addition, patients who did not visit medical institutions within 30 days or did not have information about medical institutions were excluded according to the cancer-specific insurance claim code (V193). Finally, the data of 2879 gastric cancer patients were used in this study.

### Variables

The outcome variable was five-year mortality after gastric cancer diagnosis. We defined the first date of visiting the hospital due to major diagnosis of gastric cancer as the index date and observed each patient for a maximum of 5 years (1825 days). If patients died within 5 years, they fell within the “died” group, regardless of their cause of death, and the remainder fell within the “survivor” group.

The primary variable of interest that we sought to examine regarding the association between fragmentated cancer care and five-year mortality was a change in the most visited hospital within the first year after diagnosis. Fragmented care is generally defined as when patients visit multiple medical institutions to receive care. Nevertheless, the Korean NHI manages the quality of care according to the results of the Healthcare Quality Assessment; for cancer care, the Health Insurance Review and Assessment (HIRA) is in charge of quality assessments, and one of its quality indicators is that the treatment for cancer patients should be provided within 30 days after the first diagnosis [19]. Accordingly, first, we summarized the medical costs of each medical institution within 30 days of diagnosis, and the hospital with the highest portion of medical expenses was defined as the major treatment institution. Second, we similarly defined the most visited hospital during the 31–365 days after diagnosis. If the major visiting institution changed in the period of 31–365 days, the patients fell into the “fragmented cancer care” group.

We also included other independent variables, namely sex, age (≤ 49, 50–59, 60–69, 70–79, or ≥ 80 years), type of insurance coverage, economic status, residence area (capital area, metropolitan, rural), Charlson Comorbidity Index (CCI), year of diagnosis, type of treatment within the first year, and type or location of the major treatment institution. Regarding the classification of the Korean NHI coverage, around 97% of individuals were NHI beneficiaries, and were classified into the NHI employee (all employees and employers whose household members were also covered) and NHI self-employed (all other individuals, who had insurance premiums calculated based on income, property, and living standards) groups. The remaining 3% consisted of the Medical-Aid group, comprising individuals with low income or disabilities who did not pay insurance premiums. Typically, NHI beneficiaries only pay a 5% co-payment for medical costs associated with cancer care, while the Medical-Aid group pays 0% of inpatient care and 0–5% of outpatient care costs.

Economic status was calculated using the insurance premium, which was in turn paid according to the individual’s economic level and was classified as < 30 (low), 31–60 (mid-low), 61–80 (mid), and ≥ 81 (high).

The CCI was utilized as an index of clinical severity, which was calculated based on medical and symptom records recorded after cancer diagnosis while excluding the score for the cancer itself. It was classified as 0–2, 3–5, or more than 5.

The type of treatment received within 1 year of diagnosis included surgery (total or subtotal gastrectomy or endoscopic submucosal dissection), chemotherapy, or radiotherapy. We then classified patients into three groups, namely “surgery and chemotherapy or radiotherapy,” “only surgery,” and “chemotherapy or radiotherapy.”

The major treatment institution was categorized based on its characteristics, namely type (tertiary hospital, general hospital, other) or location (capital area, metropolitan, rural).

### Statistical analysis

We first examined the frequency and percentage of fragmented cancer care and five-year mortality in the study population and conducted chi-square tests for the categorical variables. Next, Kaplan–Meier survival curves and the log-rank test were used to compare survival rates by fragmented cancer care.

We also analyzed the results of multiple logistic regression analysis using the generalized estimated equation model after controlling for independent variables to investigate the patient and institution characteristics that affected fragmented cancer care during the first year. Finally, survival analysis using the Cox proportional hazard model was conducted after controlling for all independent variables to investigate the association between fragmented cancer care during the first year and survival 5 years after diagnosis.

Subgroup analyses according to type of treatment were conducted to compare differences between groups (*p* for the interaction term [fragmented cancer care * type of treatment within 1 year after diagnosis] < .0001). We also performed sensitivity analysis using different period thresholds (60/90/120 days) and examined whether patients changed their major visiting hospital; the results were similar to those using the 30-day threshold (Supplement 1). All statistical analyses were performed using SAS statistical software version 9.4 (Cary, NC).

## Results

In this study, 2879 gastric cancer patients who received treatment within 1 year after diagnosis were included. Table 1 shows the general characteristics of the study population stratified by whether they experienced fragmented care or not. Regarding the results for changes in medical institution, among the 2879 patients with gastric cancer, 11.9% received fragmented cancer care due to changing the major treatment institution. Patients who lived in non-capital areas were more likely to experience fragmented cancer care than those living in the capital area (*p* < .0001). In addition, patients with severe clinical conditions or who had received treatment other than surgery changed hospitals more frequently (*p* < .0001). Patients who often visited smaller medical institutions (e.g., hospitals and clinics) or visited institutions located in rural areas during the 30 days after diagnosis also experienced fragmented cancer care more frequently (*p* < .05).

**Table 1:** Study population by fragmented cancer care and five-year mortality

Variables	Fragmented cancer care						Five-year mortality					
	Total	With		Without		*p*	Total	Died		Survivor		*p*
		N	%	N	%			N	%	N	%	
Fragmented cancer care												
With							342	79	23.1	263	76.9	0.0042
Without							2537	427	16.8	2110	83.2	
Sex												
Male	1935	228	11.8	1707	88.2	0.8194	1935	361	18.7	1574	81.3	0.0292
Female	944	114	12.1	830	87.9		944	145	15.4	799	84.6	
Age (Years)												
≤ 49	574	64	11.1	510	88.9	0.8798	574	85	14.8	489	85.2	<.0001
50–59	761	93	12.2	668	87.8		761	108	14.2	653	85.8	
60–69	859	99	11.5	760	88.5		859	141	16.4	718	83.6	
70–79	596	73	12.2	523	87.8		596	144	24.2	452	75.8	
≥ 80	89	13	14.6	76	85.4		89	28	31.5	61	68.5	
Type of insurance coverage												
Medical-Aid	111	13	11.7	98	88.3	0.3858	111	27	24.3	84	75.7	0.0669
NHI, Self-employed	995	107	10.8	888	89.2		995	185	18.6	810	81.4	
NHI, Employee	1773	222	12.5	1551	87.5		1773	294	16.6	1479	83.4	
Economic status												
Low	739	74	10.0	665	90.0	0.1571	739	151	20.4	588	79.6	0.1325
Mid-low	681	91	13.4	590	86.6		681	113	16.6	568	83.4	
Mid-high	608	67	11.0	541	89.0		608	101	16.6	507	83.4	
High	851	110	12.9	741	87.1		851	141	16.6	710	83.4	
Residence area												
Capital area	1170	99	8.5	1071	91.5	<.0001	1170	209	17.9	961	82.1	0.7871
Metropolitan	758	112	14.8	646	85.2		758	127	16.8	631	83.2	
Rural	951	131	13.8	820	86.2		951	170	17.9	781	82.1	
Charlson Comorbidity Index												
≤ 2	1960	200	10.2	1760	89.8	0.0003	1960	283	14.4	1677	85.6	<.0001
3–5	791	121	15.3	670	84.7		791	182	23.0	609	77.0	
> 5	128	21	16.4	107	83.6		128	41	32.0	87	68.0	
Year of diagnosis												
2005	116	10	8.6	106	91.4	0.8692	116	21	18.1	95	81.9	<.0001
2006	289	31	10.7	258	89.3		289	70	24.2	219	75.8	
2007	299	33	11.0	266	89.0		299	64	21.4	235	78.6	
2008	307	40	13.0	267	87.0		307	60	19.5	247	80.5	
2009	358	46	12.8	312	87.2		358	81	22.6	277	77.4	
2010	306	42	13.7	264	86.3		306	64	20.9	242	79.1	
2011	386	42	10.9	344	89.1		386	59	15.3	327	84.7	
2012	385	47	12.2	338	87.8		385	36	9.4	349	90.6	
2013	433	51	11.8	382	88.2		433	51	11.8	382	88.2	
Type of treatment within 1 year after diagnosis												
Surgery and chemotherapy or radiotherapy	664	78	11.7	586	88.3	0.0007	664	240	36.1	424	63.9	<.0001
Only surgery	2056	230	11.2	1826	88.8		2056	145	7.1	1911	92.9	
Chemotherapy or radiotherapy	159	34	21.4	125	78.6		159	121	76.1	38	23.9	
Type of most visited medical institution within 1 month												
Tertiary hospital	1990	210	10.6	1780	89.4	<.0001	1990	336	16.9	1654	83.1	0.0686
General hospital	852	115	13.5	737	86.5		852	167	19.6	685	80.4	
Other	37	17	45.9	20	54.1		37	3	8.1	34	91.9	
Location of most visited medical institution within 1 month												
Capital area	1688	174	10.3	1514	89.7	0.0071	1688	301	17.8	1387	82.2	0.5420
Metropolitan	792	109	13.8	683	86.2		792	130	16.4	662	83.6	
Rural	399	59	14.8	340	85.2		399	75	18.8	324	81.2	
Total	2879	342	11.9	2537	88.1		2879	506	17.6	2373	82.4	

Regarding mortality, 17.6% of patients died within 5 years after gastric cancer diagnosis; a higher number of patients who experienced fragmented cancer care during the first year after diagnosis (23.1% vs. others: 16.8%; *p* < .0001), male patients (18.7% vs. female patients: 15.4%, respectively; *p* < .0292), and older patients died (vs. younger patients; *p* < .0001). Regarding clinical characteristics, patients with higher CCI or those who did not receive surgical treatment were associated with higher mortality within 5 years (*p* < .0001). Regarding type or location of the major treatment institution, there were no statistically significant differences between groups.

Table 2 shows the results of logistic regression analysis for changes in medical institution adjusted for independent variables. There were some significant associations with fragmented cancer care. Considering type of insurance coverage, NHI self-employed patients experienced less fragmented cancer care than their NHI employed counterparts. In addition, patients with low socioeconomic status changed their medical institution less during the first year. However, patients who lived in metropolitan or rural areas experienced more fragmented cancer care within 30 days of diagnosis compared to those in the capital area (metropolitan, RR = 2.031, 95% CI = 1.373–3.003, *p* = .0004; rural, RR = 1.976, 95% CI = 1.407–2.776, *p* < .0001; ref. = capital area). In addition, patients with higher clinical severity changed medical institutions more often than those with low clinical severity (CCI 3–5, RR = 1.623, 95% CI = 1.256–2.097, *p* = .0002; CCI > 5, RR = 1.868, 95% CI = 1.114–3.133, *p* = .0179; ref. = CCI ≤2). Regarding treatment type, patients who did not receive surgical treatment but received chemotherapy or radiotherapy after diagnosis experienced more fragmented cancer care, as did patients who visited smaller medical institutions (e.g., hospitals or clinics) rather than general or tertiary hospitals.

**Table 2:** Results of logistic regression analysis for fragmented cancer care

Variables	Fragmented cancer care							
	Unadjusted				Adjusted†			
	RR	95% CI		*p*	RR	95% CI		*p*
Sex								
Male	0.972	0.765	1.236	0.8194	1.135	0.728	1.196	0.5832
Female	1.000	–	–	–	1.000	–	–	–
Age (Years)								
≤ 49	1.000	–	–	–	1.000	–	–	–
50–59	1.189	0.791	1.556	0.5477	1.036	0.730	1.470	0.8422
60–69	1.186	0.743	1.449	0.8265	0.905	0.635	1.288	0.5788
70–79	1.200	0.778	1.590	0.5592	0.886	0.605	1.299	0.5357
≥ 80	1.388	0.716	2.593	0.3452	0.971	0.488	1.930	0.9322
Type of insurance coverage								
Medical-Aid	0.927	0.511	1.681	0.8023	1.064	0.553	2.046	0.8527
NHI, Self-employed	0.842	0.659	1.076	0.1684	0.772	0.598	0.995	0.0457
NHI, Employee	1.000	–	–	–	1.000	–	–	–
Economic status								
Low	0.750	0.548	1.025	0.0709	0.670	0.475	0.945	0.0226
Mid-low	1.039	0.771	1.400	0.8014	1.033	0.759	1.407	0.8344
Mid-high	0.834	0.604	1.153	0.2720	0.783	0.560	1.093	0.1506
High	1.000	–	–	–	1.000	–	–	–
Residence area								
Capital area	1.000	–	–	–	1.000	–	–	–
Metropolitan	1.876	1.407	2.500	<.0001	2.031	1.373	3.003	0.0004
Rural	1.728	1.311	2.278	0.0001	1.976	1.407	2.776	<.0001
Charlson Comorbidity Index								
≥ 2	1.000	–	–	–	1.000	–	–	–
3–5	1.589	1.247	2.026	0.0002	1.623	1.256	2.097	0.0002
> 5	1.727	1.058	2.820	0.0289	1.868	1.114	3.133	0.0179
Year of diagnosis	1.015	0.969	1.063	0.5302	1.023	0.975	1.074	0.3539
Type of treatment within 1 year after diagnosis								
Surgery and chemotherapy or radiotherapy	1.000	–	–	–	1.000	–	–	–
Only surgery	0.946	0.720	1.244	0.6921	0.999	0.752	1.329	0.9969
Chemotherapy or radiotherapy	2.044	1.307	3.194	0.0017	2.501	1.572	3.978	0.0001
Type of most visited medical institution within 1 month								
Tertiary hospital	1.000	–	–	–	1.000	–	–	–
General hospital	1.323	1.037	1.686	0.0241	1.343	1.040	1.735	0.0238
Other	7.204	3.716	13.970	<.0001	9.128	4.598	18.116	<.0001
Location of most visited medical institution within 1 month								
Capital area	1.000	–	–	–	1.000	–	–	–
Metropolitan	1.389	1.075	1.794	0.0119	0.901	0.634	1.280	0.5598
Rural	1.510	1.099	2.075	0.0111	0.980	0.665	1.444	0.9167

† The results of the multiple logistic regression analysis using the Generalized Estimated Equation model presented herein are controlled for the covariates of: sex, age, type of insurance coverage, economic status, residence area, Charlson Comorbidity Index, year of diagnosis, type of treatment within the first year, and type or location of the medical institution which the patient visited within 1 month after diagnosis and with the highest portion of medical expenses.

**Fig. 2:**
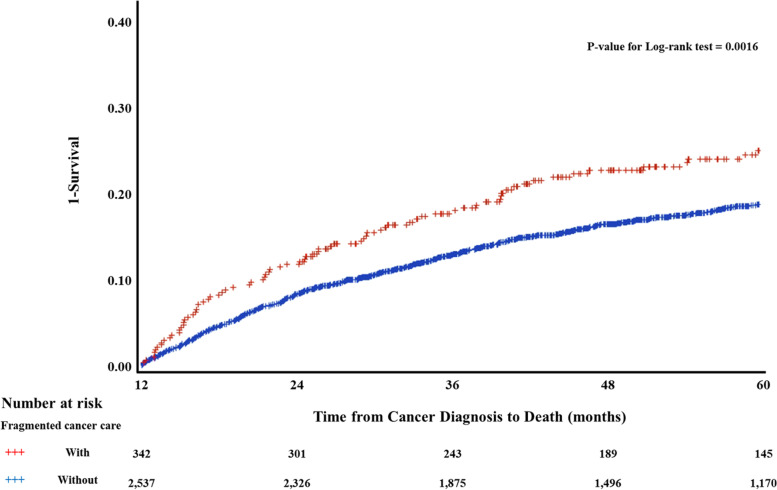
Kaplan–Meier survival curves for five-year mortality by fragmented cancer cares

Figure 2 shows the results of Kaplan–Meier survival curves and the log-rank test. Compared to patients with fragmented cancer care who changed their most visited medical institution within 30 days after diagnosis, those who changed between 31 and 365 days had a longer survival period (survival period; changed, M = 1398.1, SD = 480.7; unchanged, M = 1449.5, SD = 445.9; log-rank test, *p* = .0016).

Table 3 shows the results of survival analysis using the Cox proportional hazard model to investigate the associations of variables of interest with five-year mortality. Compared to patients with fragmented cancer care who changed their most visited medical institution within 30 days after diagnosis, those who changed between 31 and 365 days were at higher risk of mortality within 5 years (HR = 1.310, 95% CI = 1.023–1.677, *p* < .0323; ref. = unchanged). Male or older patients were also associated with higher mortality. Regarding insurance and economic status type, there were no significant associations with mortality. However, CCI (i.e., patient clinical status index) was positively associated with higher mortality (CCI 3–5, HR = 1.487, 95% CI = 1.225–1.805, *p* < .0001; CCI > 5, HR = 1.777, 95% CI = 1.262–2.502, *p* = .0010; ref. = CCI ≤ 2). Patients who received only surgery had a lower risk of mortality within 5 years than patients who received both surgery and chemotherapy or radiotherapy (only surgery, HR = 0.163, 95% CI = 0.132–0.201, *p* < .0001; ref. = surgery and chemotherapy or radiotherapy), but patients who only received chemotherapy or radiotherapy had a higher risk of mortality (chemotherapy or radiotherapy, HR = 3.710, 95% CI = 2.952–4.663, *p* < .0001; ref. = surgery and chemotherapy or radiotherapy).

**Table 3:** Results of survival analysis to identify the association between fragmented cancer care and five-year mortality

Variables	Five-year mortality							
	Unadjusted				Adjusted†			
	HR	95% CI		*P*-value	HR	95% CI		*P*-value
Fragmented cancer care								
With	1.431	1.125	1.819	0.0035	1.310	1.023	1.677	0.0323
Without	1.000	–	–	–	1.000	–	–	–
Sex								
Male	1.229	1.014	1.490	0.0360	1.250	1.026	1.523	0.0267
Female	1.000	–	–	–	1.000	–	–	–
Age (Years)								
≤ 49	1.000	–	–	–	1.000	–	–	–
50–59	0.990	0.745	1.316	0.9458	0.798	0.598	1.066	0.1269
60–69	1.137	0.868	1.488	0.3515	0.960	0.723	1.274	0.7756
70–79	1.790	1.369	2.341	<.0001	1.421	1.069	1.890	0.0157
≥ 80	2.640	1.722	4.048	<.0001	2.305	1.456	3.651	0.0004
Type of insurance coverage								
Medical-Aid	1.492	1.006	2.212	0.0468	1.081	0.699	1.671	0.7274
NHI, Self-employed	1.133	0.943	1.362	0.1834	1.141	0.945	1.377	0.1715
NHI, Employee	1.000	–	–	–	1.000	–	–	–
Economic status								
Low	1.244	0.989	1.565	0.0622	1.099	0.858	1.406	0.4554
Mid-low	0.992	0.775	1.271	0.9500	0.922	0.718	1.185	0.5264
Mid-high	0.990	0.767	1.278	0.9382	0.850	0.656	1.100	0.2166
High	1.000	–	–	–	1.000	–	–	–
Residence area								
Capital area	1.000	–	–	–	1.000	–	–	–
Metropolitan	0.912	0.732	1.137	0.4143	1.055	0.773	1.442	0.7347
Rural	0.986	0.806	1.208	0.8933	1.173	0.908	1.514	0.2222
Charlson Comorbidity Index								
≥ 2	1.000	–	–	–	1.000	–	–	–
3–5	1.702	1.412	2.050	<.0001	1.487	1.225	1.805	<.0001
> 5	2.538	1.829	3.522	<.0001	1.777	1.262	2.502	0.0010
Year of diagnosis	0.950	0.915	0.987	0.0081	1.011	0.972	1.051	0.5951
Type of treatment within 1 year after diagnosis								
Surgery and chemotherapy or radiotherapy	1.000	–	–	–	1.000	–	–	–
Only surgery	0.170	0.138	0.209	<.0001	0.163	0.132	0.201	<.0001
Chemotherapy or radiotherapy	4.009	3.214	5.000	<.0001	3.710	2.952	4.663	<.0001
Type of most visited medical institution within 1 month								
Tertiary hospital	1.000	–	–	–	1.000	–	–	–
General hospital	1.108	0.976	1.414	0.0892	1.056	0.871	1.281	0.5796
Other	0.434	0.139	1.351	0.1497	0.345	0.109	1.091	0.0700
Location of most visited medical institution within 1 month								
Capital area	1.000	–	–	–	1.000	–	–	–
Metropolitan	0.897	0.730	1.102	0.2998	0.957	0.713	1.283	0.7669
Rural	1.043	0.810	1.343	0.7461	0.861	0.630	1.176	0.3457

† The results of survival analysis using the Cox proportional hazard model was conducted after controlling for the covariates of: sex, age, type of insurance coverage, economic status, residence area, Charlson Comorbidity Index, year of diagnosis, type of treatment within the first year, and type or location of the medical institution which the patient visited within 1 month after diagnosis and with the highest portion of medical expenses.

**Fig. 3:**
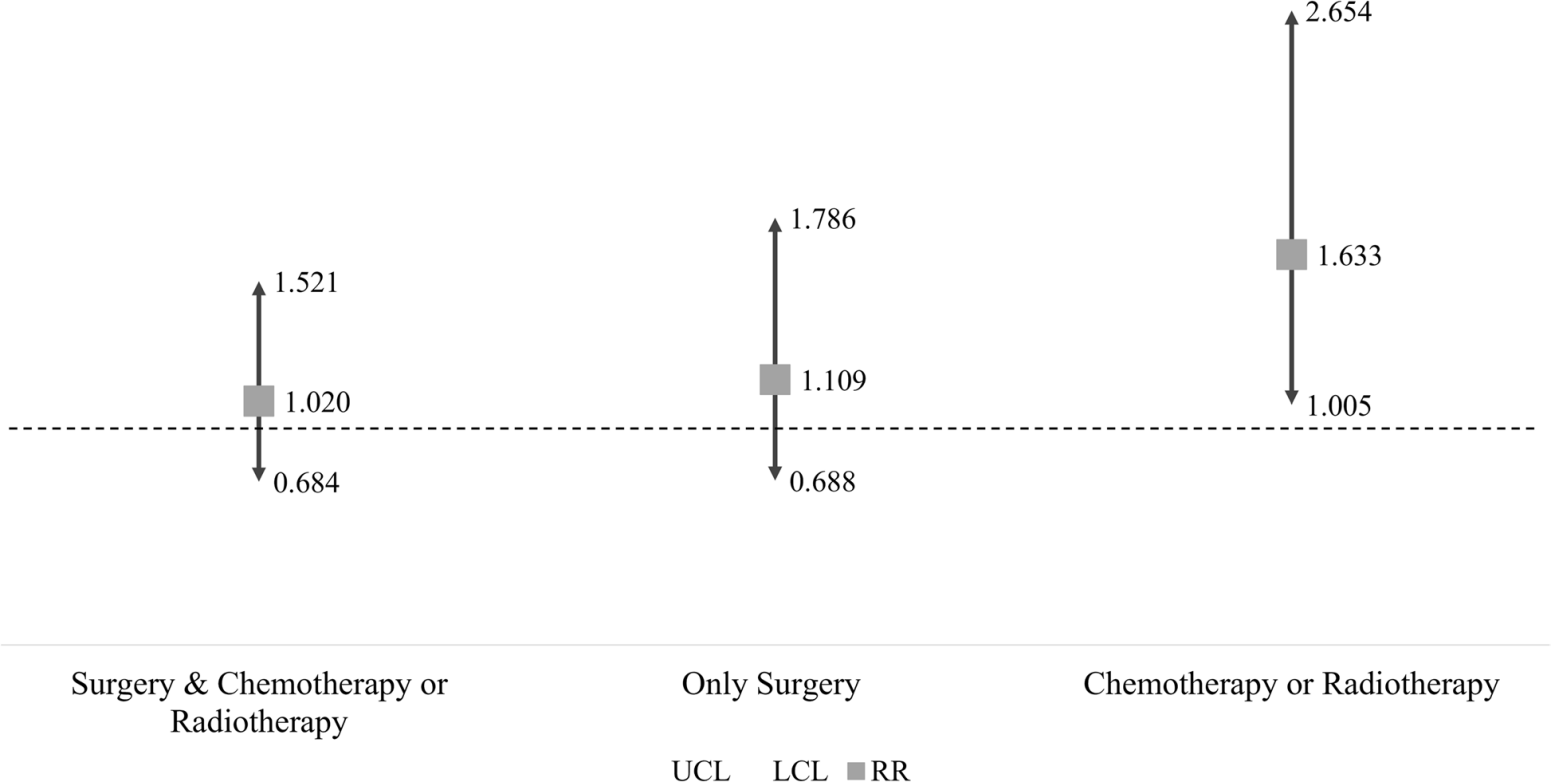
Results of survival analysis according to treatment type within 1 year of diagnosis. * If the arrow on the box plot meets the dotted line, the result is not statistically significant (*P*-value > 0.05)

In addition, we performed a subgroup analysis for survival according to the treatment type provided to patients within the first year after diagnosis. Interaction associations between fragmented cancer care and types of treatment were present. For patients who received surgical treatment with or without other forms of therapy, there was no statistically significant association with mortality within 5 years, but there were positive trends. However, among patients who received only chemotherapy or radiotherapy, fragmented cancer care had a statistically significant association with higher mortality (HR: 1.633, 95% CI: 1.005–2.654, *P*-value: 0.0477; Fig. 3).

## Discussion

In this study, we analyzed the association between the survival of gastric cancer patients and fragmented cancer care, with fragmented cancer care being defined as changes to patients’ most visited medical institutions either within 1 month of diagnosis or in the period between 2 months and 1 year of diagnosis. We observed that fragmented cancer care was associated with worsening patient outcomes, and that changes showed up more frequently in either patients with severe conditions or who mainly visited smaller medical institutions in the first month after diagnosis.

Previous studies have shown that patients who visit hospitals for surgical treatment concomitantly receiving cancer treatment at other local hospitals may experience more changes in medical institutions; this is because they are more likely to want more sophisticated oncology care, to transfer to high-volume hospitals, or they may not be satisfied with the standard of cancer care in the hospital they had initially been visiting [6, 16]. Similarly, our study showed that patients in metropolitan or rural areas changed their most visited medical institution more than those in the capital area, wherein there are more high-volume hospitals. This finding is consistent with known barriers to cancer treatment in rural communities, namely limited access to doctors providing cancer screening and treatment and geographic distance to healthcare facilities [20, 21]. Therefore, this result raises important concerns regarding a potential imbalance in the Korean cancer care delivery system across different areas, as well as its concentration in the capital area.

Although fragmented cancer care may be associated with unnecessary and redundant services, low patient satisfaction, and low treatment effects, it is still unclear whether these associations translate into treatment timing or overall survival [6, 12, 22–24]. Moreover, because of the complexity of cancer care, the implications of fragmented care delivery may be exacerbated and may fuel healthcare spending for patients, providers, and insurers [6]. In a previous hepatocellular carcinoma study, it was found that fragmented cancer care was independently associated with increased time until the commencement of treatment and decreased overall survival [4]. Other authors further indicated that, in comparison to surgeons in low-volume hospitals, those in high-volume hospitals are more likely to collaborate in decisions about adjuvant chemotherapy with oncologists within their institution, and patients may prefer to remain in a high-volume cancer center for their medical oncology care [16, 25]. In particular, the most important consequence of delays caused by transfer of care is that the time between diagnosis and the commencement of oncology treatments, such as chemotherapy and radiation therapy, may be directly lengthened [26]. As a result, this may lead to a higher risk of mortality for patients who have changed medical institutions compared to those who have not. The results of subgroup analysis showed that patients who received chemotherapy or radiation therapy, excluding surgical treatment, had a greater association with mortality according to the fragmented cancer care. Therefore, carefully examining the symptoms of patients with advanced or terminal gastric cancer, who need chemotherapy or radiotherapy, based on the continuity of care is necessary.

However, a study conducted by Hussain et al. on fragmented care for patients with colorectal cancer did not find an association between fragmented care and overall survival [16]. Furthermore, they indicated that adjuvant therapy has been shown to improve the overall survival of stage 3 colorectal cancer patients [16], and that it is currently recommended by the U.S. Comprehensive Cancer Network Guidelines [27, 28]. The limitation of coordination failure associated with neoadjuvant and adjuvant therapy can also significantly bias survival data [28]. In contrast to the study by Hussain et al., fragmented cancer care was associated with worsened survival in the current study. A potential explanation may be related to the differences in study design and healthcare systems between studies, and another is that we analyzed all types of gastric cancer, whereas their study provides findings only for advanced cancer types.

Our findings provide several policy implications. First, most Korean patients currently rely on the reputation or size of the medical institution when choosing where to get treatment, which implies that they generally do not fully consider their residency nor the severity of their illness during related decision-making. Thus, policymakers should review related policies in order to ensure the provision of a more efficient decision-making assistance service for patients regarding which medical institution to visit to receive care when they need it. Second, it may be that some patients wonder which institution they should seek to receive secondary care after they receive aggressive cancer care, which often occurs in the capital area and is one of the situations related to the aforementioned concentration of patients in this area. Therefore, a community-based patient linkage system could be constructed to guide patients to seek care in their community after they receive aggressive cancer care.

This study has several limitations. First, in this nationwide sampling cohort based on claims data, information regarding clinical test results and the severity of cancer were not collected due to the lack of detailed clinical information. Second, considering the nature of retrospective data based on claims, the findings presented in this study cannot be used to establish causal associations. Therefore, our results should be interpreted with care and may not be generalizable to settings beyond Korea. Third, this was an observational study, not a randomized trial, so we could not fully adjust for hidden bias. Fourth, although administrative databases are increasingly used for clinical research, these studies are potentially vulnerable to measurement errors caused by incorrect coding. Fifth, although we adjusted for CCI to account for disease severity, this index does not provide a thorough consideration of the health conditions of patients (e.g., it does not account for cancer stage), and we also could not analyze such data due to the limitations inherent to the administrative data set used (i.e., on medical cost reimbursement claims).

## Conclusions

This study suggests that fragmented cancer care was associated with increased risk of five-year mortality, and that changes in the most visited institution occurred more frequently in patients who either had severe conditions or who mainly visited smaller medical institutions in the first month after diagnosis. Despite these significant associations, there is still lack of consensus across the existing literature. Further study is warranted to confirm these findings and examine a causal relationship between fragmented cancer care and survival.

## Supplementary Information

**Additional file 1:** **Supplementary Table 1.** The results of sensitivity analysis according to different period thresholds. † The results of survival analysis using the Cox proportional hazard model was conducted after controlling for the covariates of: sex, age, type of insurance coverage, economic status, residence area, Charlson Comorbidity Index, year of diagnosis, type of treatment within the first year, and type or location of the medical institution which the patient visited within 1 month after diagnosis and with the highest portion of medical expenses.

## Data Availability

The data that support the findings of this study are National Health Insurance Service claims data and are stored on a separate server managed by the National Health Insurance Service. The datasets generated and analyzed during the current study are not publicly available due to restrictions imposed by the National Health Insurance Service. Data are available from the corresponding author upon reasonable request and with permission from the National Health Insurance Service.

## Abbreviations

CCI: Charlson Comorbidity Index
HIRA: Health Insurance Review and Assessment
ICD: International Classification of Diseases
NHI: National Health Insurance
