# Supplementary material for: Does fragmented cancer care affect survival? Analysis of gastric cancer patients using national insurance claim data
Source: BMC Health Serv Res. 2022 Dec 21;22:1566. doi: 10.1186/s12913-022-08988-y (PMC9773508; doi:10.1186/s12913-022-08988-y)
Supplement: Supplementary file 1 — Additional file 1: Supplementary Table 1. The results of sensitivity analysis according to different period thresholds. † The results of survival analysis using the Cox proportional hazard model was conducted after controlling for the covariates of: sex, age, type of insurance coverage, economic status, residence area, Charlson Comorbidity Index, year of diagnosis, type of treatment within the first year, and type or location of the medical institution which the patient visited within 1 month after diagnosis and with the highest portion of medical expenses. [file 12913_2022_8988_MOESM1_ESM.docx]

**Supplementary table 1. The results of sensitivity analysis according to different period thresholds**

| **Variables** | **Five-year mortality** | | | |
| --- | --- | --- | --- | --- |
|  | **HR** | **95% CI** | | **P-value** |
| **Fragmented cancer care (30 days vs. 31–365 days)** |  |  |  |  |
| With | 1.310 | 1.023 | 1.677 | 0.0323 |
| Without | 1.000 | - | - | - |
| **Fragmented cancer care (60 days vs. 61–365 days)** |  |  |  |  |
| With | 1.455 | 1.127 | 1.879 | 0.0041 |
| Without | 1.000 | - | - | - |
| **Fragmented cancer care (90 days vs. 91–365 days)** |  |  |  |  |
| With | 1.674 | 1.296 | 2.163 | <.0001 |
| Without | 1.000 | - | - | - |
| **Fragmented cancer care (120 days vs. 121–365 days)** |  |  |  |  |
| With | 1.677 | 1.289 | 2.181 | 0.0001 |
| Without | 1.000 | - | - | - |

† The results of survival analysis using the Cox proportional hazard model was conducted after controlling for the covariates of: sex, age, type of insurance coverage, economic status, residence area, Charlson Comorbidity Index, year of diagnosis, type of treatment within the first year, and type or location of the medical institution which the patient visited within one month after diagnosis and with the highest portion of medical expenses.
